# ﻿Multigene phylogeny and morphology reveal three new species of *Cytospora* isolated from diseased plant branches in Fengtai District, Beijing, China

**DOI:** 10.3897/mycokeys.101.116272

**Published:** 2024-01-18

**Authors:** Aoli Jia, Baoyue Chen, Hongyan Lu, Yu Xing, Bin Li, Xinlei Fan

**Affiliations:** 1 State Key Laboratory of Efficient Production of Forest Resources, Beijing Forestry University, Beijing 100083, China; 2 Key Laboratory for Silviculture and Conservation of the Ministry of Education, Beijing Forestry University, Beijing 100083, China; 3 Forestry Workstation, Fengtai District Bureau of Forestry and Parks of Beijing Municipality, Beijing 100055, China

**Keywords:** Canker disease, Diaporthales, pathogens, taxonomy

## Abstract

Members of *Cytospora* include saprobes, endophytes and important plant pathogens, which are widely distributed on various wood hosts and have a wide global distribution. In this study, the species definitions were conducted, based on multigene phylogeny (ITS, *act*, *rpb2*, *tef1-α* and *tub2* genes) and comparisons of morphological characters. A total of 22 representative isolates obtained from 21 specimens in Fengtai District of Beijing City were identified as seven species of *Cytospora*, including four known species (*C.albodisca*, *C.ailanthicola*, *C.euonymina*, *C.haidianensis*) and three novel species (*C.fengtaiensis*, *C.pinea*, *C.sorbariae*). The results provide an understanding of the taxonomy of *Cytospora* species associated with canker and dieback diseases in Fengtai District, Beijing, China.

## ﻿Introduction

The genus *Cytospora* was established by Ehrenberg (1818) and classified in Cytosporaceae, Diaporthales, Sordariomycetes (Wijayawardene et al. 2018; Fan et al. 2020). It includes numerous important pathogens associated with canker and dieback diseases of woody plants, with a worldwide distribution and broad host range (Sinclair et al. 1987; Adams et al. 2005, 2006; Lawrence et al. 2018; Fan et al. 2020; Lin et al. 2023a, b). Dieback and stem canker caused by *Cytospora* lead to the growth weakness or death of host plants, thereby causing significant economic and ecological losses (Sinclair et al. 1987; Adams et al. 2005). Currently, 695 species epithets of *Cytospora* have been listed in Index Fungorum (www.indexfungorum.org; accessed on 24 November 2023).

The taxonomy and correspondence between sexual and asexual morphs of *Cytospora* is quite confusing. Previous *Cytospora* species and their related sexual morphs viz. *Leucostoma*, *Valsa*, *Valsella* and *Valseutypella* were listed by old fungal literature for their identification (Fries 1823; Saccardo 1884; Kobayashi 1970; Barr 1978; Sutton 1980; Gvritishvili 1982; Spielman 1983, 1985). Adams et al. (2005) revised the genus *Cytospora* from *Eucalyptus* with 28 species and accepted all sexual genera combined under *Valsa*, either as subgenera or species without additional infrageneric rank, regarding the sexual genera (*Leucocytospora*, *Leucostoma*, *Valsella* and *Valseutypella*) as synonyms of *Valsa*. Based on the one fungus = one name initiative (Wingfield et al. 2012), Fan et al. (2015a, b) and Rossman et al. (2015) recommended to use *Cytospora*, the oldest name having priority over *Valsa*.

*Cytospora* canker symptoms initially appear on trunks and branches as slightly sunken bark with brown discolouration of the xylem, which may result in trunk and branch cracking (Adams et al. 2005). The asexual morph of *Cytospora* is characterised by the pycnidial stromata submerged in cortex with single or multiple locule(s), with or without conceptacle, filamentous conidiophores producing hyaline, allantoid, eguttulate and smooth conidia. The sexual morph is characterised by the ascomata submerged in the substrate with an erumpent pseudostroma, with or without necks. Asci are unitunicate, clavate to cylindrical with four or eight ascospores which are biseriate or multi-seriate, elongate-allantoid, thin-walled, hyaline and aseptate (Spielman 1983, 1985; Adams et al. 2005).

Currently, use of polyphasic approaches, such as morphological and phylogenetic analyses to define species of *Cytospora* has been proposed (Norphanphoun et al. 2017; Fan et al. 2020). In morphology, presence or absence of conceptacle, quantity and arrangement of locule(s), shape and size of conidiophores and conidial size are significantly taxonomic. In phylogeny, the current studies use the internal transcribed spacer (ITS), the partial actin (*act*), the RNA polymerase II subunit (*rpb2*), the translation elongation factor *1-α* (*tef1-α*) and the beta-tubulin (*tub2*) genes to perform phylogenetic analysis.

Beijing is the capital city of China, located in the northern part of the North China Plain with more than 1,000 species of tree hosts (Liu et al. 2022). As more plant species were recorded in this city, the exploration of fungal diversity gradually increased as most fungi are often linked to particular host plants as pathogens or endophytes. With the modern taxonomic approaches applying, more than 30 *Cytospora* species have been reported in the last five years in Beijing (Fan et al. 2020; Pan et al. 2021; Lin et al. 2023a, b). Fengtai is one of the districts in Beijing with high forest cover and rich tree species which is located in the south-western suburbs of Beijing. However, there are few studies associated with fungal diversity in Fengtai District. A research to explore more hidden species of *Cytospora* in this region is considered imperative. Therefore, a survey on the diversity of *Cytospora* on diseased branches was conducted in Fengtai District from 2022 to 2023. The objectives of this study were to summarise the systematic study of *Cytospora* species in Fengtai District and to clarify the systematics and taxonomy of *Cytospora* species with detailed descriptions and illustrations and compare it to known species in the genus.

## ﻿Materials and methods

### ﻿Sample collection and isolation

**Figure 1.**
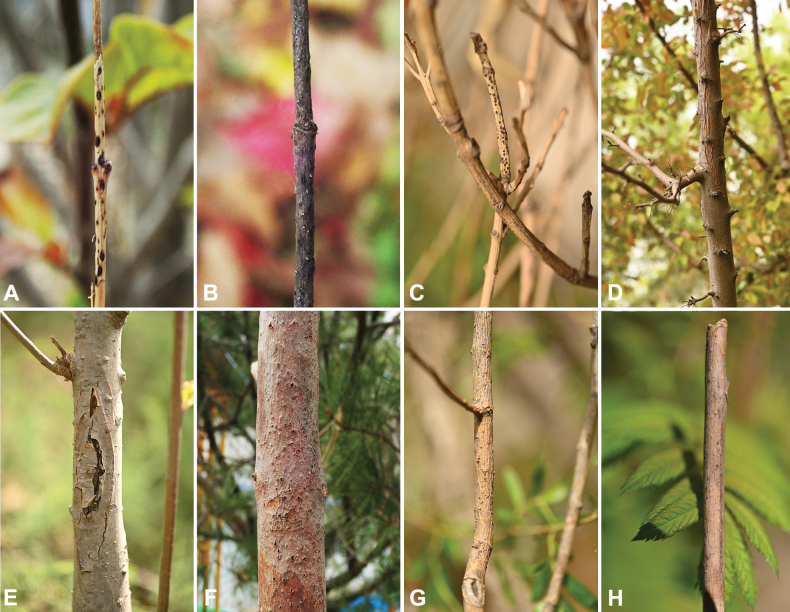
Disease symptoms associated with *Cytospora* species collected from Fengtai District, Beijing, China **A***Acerpalmatum* ‘*Atropurpureum*’ **B***Acerpictum* subsp. *Mono*. **C***Euonymusjaponicus***D***Malus* ‘*American*’ **E**Malus×micromalus**F***Pinusbungeanae***G***Salixbabylonica***H***Sorbariasorbifolia*.

Twenty-one fresh specimens with typical conidiomata and/or ascomata were collected from diseased branches or twigs of wood hosts which are distributed in Beigong National Forest Park, Century Forest Park, Garden Expo Park, Lotus Pond Park and Qianling Mountain in Fengtai District, Beijing City. Sampled trees expressed general symptoms and signs of canker diseases including elongate, slightly sunken and discoloured areas in the bark, several prominent dark conidiomata and/or ascomata immersed in bark, erumpent through the surface of bark when mature (Fig. 1). A total of 22 isolates were obtained by removing a mucoid spore mass from conidiomata and/or ascomata, spreading the suspension on the surface of 1.8% potato dextrose agar (PDA) (potato, 200 g; glucose, 20 g; agar, 20 g; distilled water, to complete 1000 ml) media in a Petri dish and incubating at 25 °C for up to 24 h. Hyphal tips were removed to a new PDA plate twice to obtain a pure culture. Specimens were deposited in the Museum of Beijing Forestry University (BJFC) and at the working Collection of X.L. Fan (CF), housed at the BJFU. Axenic cultures are maintained in the China Forestry Culture Collection Centre (CFCC).

### ﻿Morphological analyses

The identification of species was based on morphological characteristics of the ascomata or conidiomata formed on infected host materials. Macro-morphological features (structure and size of conidiomata and ascomata, ectostromatic disc and ostioles) were photographed using a Leica stereomicroscope (M205 FA) (Leica Microsystems, Wetzlar, Germany). Micromorphological features (conidiophores, conidiogenous cells, asci and conidia/ascospores) were photographed using a Nikon Eclipse 80i microscope (Nikon Corporation, Tokyo, Japan), equipped with a Nikon digital sight DS-Ri2 high resolution colour camera with differential interference contrast. Over 30 conidiomata were sectioned and 50 conidia were selected randomly to measure their lengths and widths. Colony diameters were measured and the colony colours described after 3 days and 14 days according to the colour charts of Rayner (1970).

### ﻿DNA extraction, PCR amplification and sequencing

Mycelium used for DNA extraction was grown on PDA for three days and obtained from the cellophane surface by scraping. The genomic DNA was extracted using the modified CTAB method (Doyle and Doyle 1990). PCR amplifications and sequencing of five genes (ITS, *act*, *rpb2*, *tef1-α* and *tub2*) were performed. The primers and PCR conditions are listed in Table 1. PCR products were electrophoresed in 1% agarose gel and the DNA was sequenced by the Sino Geno Max Biotechnology Company Limited (Beijing, China). DNA sequences generated by the forward and reverse primers combination were used to obtain consensus sequences using Seqman v. 7.1.0 (DNASTAR Inc., Madison, WI, USA).

**Table 1.** Genes used in this study with PCR primers, primer DNA sequence, optimal annealing temperature and corresponding references.

Locus	PCR primers	PCR: thermal cycles: (Annealing temp. in bold)	References of primers used
ITS	ITS1	(95 °C: 30 s, 51 °C: 30 s, 72 °C: 1min) × 35 cycles	White et al. (1990)
	ITS4		
*act*	ACT-512F	(95 °C: 45 s, 55 °C: 45 s, 72 °C: 1min) × 35 cycles	Carbone and Kohn (1999)
	ACT-783R		
*rpb2*	RPB2-5F	(95 °C: 30 s, 52 °C: 1 min, 72 °C:1 min) × 35 cycles	Liu et al. (1999)
	RPB2-7cR		
*tef1-α*	728F	(95 °C: 15 s, 55 °C: 20 s, 72 °C: 1min) × 35 cycles	Rehner et al. (2005)
	1567R		
*tub2*	T1	(95 °C: 30 s, 55 °C: 30 s, 72 °C: 1min) × 35 cycles	Glass and Donaldson (1995)
	Bt2b		

### ﻿Phylogenetic analyses

The phylogenetic analyses were performed, based on the individual datasets of each gene region and combined five genes (ITS, *act*, *rpb2*, *tef1-α* and *tub2*) to compare *Cytospora* species from the current study with other sequences obtained from GenBank. The sequence datasets used in this study were based on Lin et al. (2023b). Sequence alignments of the individual gene were performed in MAFFT v. 6 (Katoh and Standley 2013) and adjusted by MEGA v. 6.0 (Tamura et al. 2013). Ambiguous regions were excluded from alignments. Phylogenetic analyses were conducted using the programme PhyML v. 3.0 (Guindon et al. 2010) for Maximum Likelihood (ML) analysis and MrBayes v. 3.1.2 (Ronquist and Huelsenbeck 2003) for Bayesian Inference (BI) analysis. For ML analysis, the substitution model (GTR+G+I model) for each dataset was selected following recent studies (Fan et al. 2020; Pan et al. 2020, 2021). Confidence levels for the nodes were determined using 1,000 replicates of bootstrapping methods (Hillis and Bull 1993). For BI analysis, the best-fit evolutionary models for each partitioned locus were estimated in MrModelTest v. 2.3 (Posada and Crandall 1998) with a Markov Chain Monte Carlo algorithm. Phylograms were plotted in FigTree v. 1.4.3 (http://tree.bio.ed.ac.uk/software/figtree) and edited in Adobe Illustrator CS6 v.16.0.0 (https://www.adobe.com/cn/products/illustrator.html). Sequence data were submitted to GenBank (https://www.ncbi.nlm.nih.gov) (Table 2). The multigene sequence alignments and the trees obtained were deposited in TreeBASE (https://treebase.org; study ID S30958).

**Table 2.** Strains of *Cytospora* used in the molecular analyses in this study.

Species	Strain	Host	Origin	GenBank accession numbers				
				ITS	*act*	*rpb2*	*tef1-α*	*tub2*
*Cytosporaailanthicola*	CFCC 89970	*Ailanthusaltissima*	Ningxia, China	MH933618	MH933526	MH933592	MH933494	MH933565
***Cytosporaailanthicola***	**CFCC 59446**	***Salixmatsudana***	**Beijing, China**	**OR826163**	**OR831996**	**OR832018**	**OR832040**	**OR832062**
*Cytosporaalbodisca*	CFCC 53161	*Platycladusorientalis*	Beijing, China	MW418406	MW422899	MW422909	MW422921	MW422933
*Cytosporaalbodisca*	CFCC 54373	*Platycladusorientalis*	Beijing, China	MW418407	MW422900	MW422910	MW422922	MW422934
***Cytosporaalbodisca***	**CFCC 59467**	***Malus×micromalus***	**Beijing, China**	**OR826179**	**OR832012**	**OR832034**	**OR832056**	**OR832076**
***Cytosporaalbodisca***	**CFCC 59537**	***Euonymusjaponicus***	**Beijing, China**	**OR826180**	**OR832013**	**OR832035**	**OR832057**	**OR832077**
*Cytosporaalba*	CFCC 55462^T^	*Salixmatsudana*	Gansu, China	MZ702593	OK303457	OK303516	OK303577	OK303644
*Cytosporaalba*	CFCC 55463^T^	*Salixmatsudana*	Gansu, China	MZ702594	OK303458	OK303517	OK303578	OK303645
*Cytosporaampulliformis*	MFLUCC 16-0583^T^	*Sorbusintermedia*	Russia	KY417726	KY417692	KY417794	NA	NA
*Cytosporaampulliformis*	MFLUCC 16-0629	*Acerplatanoides*	Russia	KY417727	KY417693	KY417795	NA	NA
*Cytosporaamydgali*	CBS 144233^T^	*Prunusdulcis*	California, USA	MG971853	MG972002	NA	MG971659	MG971718
*Cytosporaatrocirrhata*	CFCC 89615	*Juglansregia*	Qinghai, China	KR045618	KF498673	KU710946	KP310858	KR045659
*Cytosporaatrocirrhata*	CFCC 89616	*Juglansregia*	Qinghai, China	KR045619	KF498674	KU710947	KP310859	KR045660
*Cytosporaatrocirrhata*	CXY 1401	*Populus* sp.	Inner Mongolia, China	JX534242	NA	NA	NA	KM034904
*Cytosporaatrocirrhata*	CXY 1402	*Populus* sp.	Inner Mongolia, China	JX534243	NA	NA	NA	KM034903
*Cytosporaavicennae*	IRAN 4199C^T^	*Malusdomestica*	Nahavand, Iran	MW295650	MZ014511	MW824358	MW394145	NA
*Cytosporaavicennae*	IRAN 4625C	*Malusdomestica*	Arak, Iran	OM368648	NA	NA	OM372510	NA
*Cytosporaazerbaijanica*	IRAN 4201C^T^	*Malusdomestica*	Urmia, Iran	MW295526	MZ014513	MW824360	MW394147	NA
*Cytosporaazerbaijanica*	IRAN 4627C	*Malusdomestica*	Miandoab, Iran	OM368650	NA	NA	OM372512	NA
*Cytosporabeilinensis*	CFCC 50493^T^	*Pinusarmandii*	Beijing, China	MH933619	MH933527	NA	MH933495	MH933561
*Cytosporabeilinensis*	CFCC 50494	*Pinusarmandii*	Beijing, China	MH933620	MH933528	NA	MH933496	MH933562
*Cytosporaberberidis*	CFCC 89927^T^	*Berberisdasystachya*	Qinghai, China	KR045620	KU710990	KU710948	KU710913	KR045661
*Cytosporaberberidis*	CFCC 89933	*Berberisdasystachya*	Qinghai, China	KR045621	KU710991	KU710949	KU710914	KR045662
*Cytosporabungeanae*	CFCC 50495^T^	*Pinusbungeanae*	Shanxi, China	MH933621	MH933529	MH933593	MH933497	MH933563
*Cytosporabungeanae*	CFCC 50496	*Pinusbungeanae*	Shanxi, China	MH933622	MH933530	MH933594	MH933498	MH933564
*Cytosporacalamicola*	MFLUCC 15-0397	*Calamus*	Thailand	NR_185736	NA	NA	ON734013	NA
*Cytosporacalifornica*	CBS 144234^T^	*Juglansregia*	California, USA	MG971935	MG972083	NA	MG971645	NA
*Cytosporacarbonacea*	CFCC 89947	*Ulmuspumila*	Qinghai, China	KR045622	KP310842	KU710950	KP310855	KP310825
*Cytosporacarpobroti*	CMW 48981^T^	*Carpobrotusedulis*	South Africa	MH382812	NA	NA	MH411212	MH411207
*Cytosporaceltidicola*	CFCC 50497^T^	*Celtissinensis*	Anhui, China	MH933623	MH933531	MH933595	MH933499	MH933566
*Cytosporaceltidicola*	CFCC 50498	*Celtissinensis*	Anhui, China	MH933624	MH933532	MH933596	MH933500	MH933567
*Cytosporacentrivillosa*	MFLUCC 16-1206^T^	*Sorbusdomestica*	Italy	MF190122	NA	MF377600	NA	NA
*Cytosporacentrivillosa*	MFLUCC 17-1660	*Sorbusdomestica*	Italy	MF190123	NA	MF377601	NA	NA
*Cytosporaceratosperma*	CFCC 89624	*Juglansregia*	Gansu, China	KR045645	NA	KU710976	KP310860	KR045686
*Cytosporaceratosperma*	CFCC 89625	*Juglansregia*	Gansu, China	KR045646	NA	KU710977	KP31086	KR045687
*Cytosporaceratospermopsis*	CFCC 89626^T^	*Juglansregia*	Shaanxi, China	KR045647	KU711011	KU710978	KU710934	KR045688
*Cytosporaceratospermopsis*	CFCC 89627	*Juglansregia*	Shaanxi, China	KR045648	KU711012	KU710979	KU710935	KR045689
*Cytosporachrysosperma*	CFCC 89629	*Salixpsammophila*	Shaanxi, China	KF765673	NA	KF765705	NA	NA
*Cytosporachrysosperma*	CFCC 89981	*Populusalba* subsp. *pyramidalis*	Gansu, China	MH933625	MH933533	MH933597	MH933501	MH933568
*Cytosporachrysosperma*	CFCC 89982	*Ulmuspumila*	Tibet, China	KP281261	KP310835	NA	KP310848	KP310818
*Cytosporacinnamomea*	CFCC 53178^T^	*Prunusarmeniaca*	Xinjiang, China	MK673054	MK673024	NA	NA	MK672970
*Cytosporacoryli*	CFCC 53162^T^	*Corylusmandshurica*	Beijing, China	MN854450	NA	MN850751	MN850758	MN861120
*Cytosporacorylina*	CFCC 54684^T^	*Corylusheterophylla*	Beijing, China	MW839861	MW815951	MW815937	MW815886	MW883969
*Cytosporacorylina*	CFCC 54685	*Corylusheterophylla*	Beijing, China	MW839862	MW815952	MW815938	MW815887	MW883970
*Cytosporacorylina*	CFCC 54686	*Corylusheterophylla*	Beijing, China	MW839863	MW815953	MW815939	MW815888	MW883971
*Cytosporacorylina*	CFCC 54687	*Corylusheterophylla*	Beijing, China	MW839864	MW815954	MW815940	MW815889	MW883972
*Cytosporacotini*	MFLUCC 14-1050^T^	*Cotinuscoggygria*	Russia	KX430142	NA	KX430144	NA	NA
*Cytosporacotoneastricola*	CF 20197027	*Cotoneaster* sp.	Tibet, China	MK673072	MK673042	MK673012	MK672958	MK672988
*Cytosporacotoneastricola*	CF 20197028	*Cotoneaster* sp.	Tibet, China	MK673073	MK673043	MK673013	MK672959	MK672989
*Cytosporacotoneastricola*	CF 20197030	*Cotoneaster* sp.	Tibet, China	MK673074	MK673044	MK673014	MK672960	MK672990
*Cytosporacotoneastricola*	CF 20197031^T^	*Cotoneaster* sp.	Tibet, China	MK673075	MK673045	MK673015	MK672961	MK672991
*Cytosporacurvata*	MFLUCC 15-0865^T^	*Salixalba*	Russia	KY417728	KY417694	KY417796	NA	NA
*Cytosporacurvispora*	CFCC 54000^T^	*Corylusheterophylla*	Beijing, China	MW839851	MW815931	MW815945	MW815880	MW883963
*Cytosporacurvispora*	CFCC 54001	*Corylusheterophylla*	Beijing, China	MW839853	MW815932	MW815946	MW815881	MW883964
*Cytosporacurvispora*	CFCC 54676	*Corylusheterophylla*	Beijing, China	MW839854	MW815933	MW815947	MW815882	MW883965
*Cytosporacurvispora*	CFCC 54677	*Corylusheterophylla*	Beijing, China	MW839855	MW815934	MW815948	MW815883	MW883966
*Cytosporacurvispora*	CFCC 54678	*Corylusheterophylla*	Beijing, China	MW839856	MW815935	MW815949	MW815884	MW883967
*Cytosporacurvispora*	CFCC 54679	*Corylusheterophylla*	Beijing, China	MW839857	MW815936	MW815950	MW815885	MW883968
*Cytosporadavidiana*	CXY 1350^T^	*Populusdavidiana*	Inner Mongolia, China	KM034870	NA	NA	NA	NA
*Cytosporadiopuiensis*	MFLUCC 18-1419^T^	Undefined wood	Chiang Mai, Thailand	MK912137	MN685819	NA	NA	NA
*Cytosporadiopuiensis*	CFCC55884	Kerriajaponicaf.pleniflora	Beijing, China	OK316819	NA	OK358569	OK358471	OK358473
*Cytosporadiopuiensis*	CFCC55885	Kerriajaponicaf.pleniflora	Beijing, China	OK316820	NA	OK358570	OK358472	OK358474
*Cytosporadiopuiensis*	CFCC 56961	*Koelreuteriapaniculata*	Beijing, China	ON376918	ON390905	ON390908	ON390914	ON390923
*Cytosporadiopuiensis*	CFCC 56970	*Koelreuteriapaniculata*	Beijing, China	ON376917	ON390904	ON390907	ON390913	ON390922
*Cytosporadiopuiensis*	CFCC 56971	*Koelreuteriapaniculata*	Beijing, China	ON376919	ON390906	NA	ON390915	NA
*Cytosporadiscotoma*	CFCC 53137^T^	*Platycladusorientalis*	Beijing, China	MW418404	MW422897	MW422907	MW422919	MW422931
*Cytosporadiscotoma*	CFCC 54368	*Platycladusorientalis*	Beijing, China	MW418405	MW422898	MW422908	MW422920	MW422932
*Cytosporadonetzica*	MFLUCC 15-0864	*Crataegusmonogyna*	Russia	KY417729	KY417695	KY417797	NA	NA
*Cytosporadonetzica*	MFLUCC 16-0574^T^	*Crataegusmonogyna*	Russia	KY417731	KY417697	KY417799	NA	NA
*Cytosporadonglingensis*	CFCC 53159^T^	*Platycladusorientalis*	Beijing, China	MW418412	MW422903	MW422915	MW422927	MW422939
*Cytosporadonglingensis*	CFCC 53160	*Platycladusorientalis*	Beijing, China	MW418414	MW422905	MW422917	MW422929	MW422941
*Cytosporadonglingensis*	CFCC 54371	*Platycladusorientalis*	Beijing, China	MW418413	MW422904	MW422916	MW422928	MW422940
*Cytosporadonglingensis*	CFCC 54372	*Platycladusorientalis*	Beijing, China	MW418415	MW422906	MW422918	MW422930	MW422942
*Cytosporaelaeagni*	CFCC 89632	*Elaeagnusangustifolia*	Ningxia, China	KR045626	KU710995	KU710955	KU710918	KR045667
*Cytosporaelaeagni*	CFCC 89633	*Elaeagnusangustifolia*	Ningxia, China	KF765677	KU710996	KU710956	KU710919	KR045668
*Cytosporaelaeagnicola*	CFCC 52882^T^	*Elaeagnusangustifolia*	Xinjiang, China	MK732341	MK732344	MK732347	NA	NA
*Cytosporaelaeagnicola*	CFCC 52883	*Elaeagnusangustifolia*	Xinjiang, China	MK732342	MK732345	MK732348	NA	NA
*Cytosporaelaeagnicola*	CFCC 52884	*Elaeagnusangustifolia*	Xinjiang, China	MK732343	MK732346	MK732349	NA	NA
*Cytosporaershadii*	IRAN 4197C	*Malusdomestica*	Nahavand, Iran	MW295510	NA	NA	MW394143	NA
*Cytosporaershadii*	IRAN 4198C^T^	*Malusdomestica*	Arak, Iran	MW295523	MZ014510	MW824357	MW394144	NA
*Cytosporaerumpens*	CFCC 50022	*Prunuspadus*	Shanxi, China	MH933627	MH933534	NA	MH933502	MH933569
*Cytosporaerumpens*	MFLUCC 16-0580^T^	Salix×fragilis	Russia	KY417733	KY417699	KY417801	NA	NA
*Cytosporaerumpens*	CFCC 53163	*Prunuspadus*	Xinjiang, China	MK673059	MK673029	MK673000	MK672948	MK672975
*Cytosporaeucalypti*	CBS 144241	*Eucalyptusglobulus*	California, USA	MG971907	MG972056	NA	MG971617	MG971772
*Cytosporaeuonymicola*	CFCC 50499^T^	*Euonymuskiautschovicus*	Shaanxi, China	MH933628	MH933535	MH933598	MH933503	MH933570
*Cytosporaeuonymicola*	CFCC 50500	*Euonymuskiautschovicus*	Shaanxi, China	MH933629	MH933536	MH933599	MH933504	MH933571
*Cytosporaeuonymina*	CFCC 89993^T^	*Euonymuskiautschovicus*	Shanxi, China	MH933630	MH933537	MH933600	MH933505	MH933590
*Cytosporaeuonymina*	CFCC 89999	*Euonymuskiautschovicus*	Shanxi, China	MH933631	MH933538	MH933601	MH933506	MH933591
***Cytosporaeuonymina***	**CFCC 59444**	***Salixbabylonica***	**Beijing, China**	**OR826164**	**OR831997**	**OR832019**	**OR832041**	**NA**
***Cytosporaeuonymina***	**CFCC 59479**	***Salixbabylonica***	**Beijing, China**	**OR826165**	**OR831998**	**OR832020**	**OR832042**	**NA**
***Cytosporafengtaiensis***	**CFCC 59442**	***Acerpalmatum* ‘*Atropurpureum***’	**Beijing, China**	**OR826166**	**OR831999**	**OR832021**	**OR832043**	**OR832063**
***Cytosporafengtaiensis***	**CFCC 59449^T^**	***Acerpalmatum* ‘*Atropurpureum***’	**Beijing, China**	**OR826167**	**OR832000**	**OR832022**	**OR832044**	**OR832064**
***Cytosporafengtaiensis***	**CFCC 59525**	***Acerpalmatum* ‘*Atropurpureum***’	**Beijing, China**	**OR826168**	**OR832001**	**OR832023**	**OR832045**	**OR832065**
***Cytosporafengtaiensis***	**CFCC 59526**	***Acerpalmatum* ‘*Atropurpureum***’	**Beijing, China**	**OR826169**	**OR832002**	**OR832024**	**OR832046**	**OR832066**
***Cytosporafengtaiensis***	**CFCC 59527**	***Acerpalmatum* ‘*Atropurpureum***’	**Beijing, China**	**OR826170**	**OR832003**	**OR832025**	**OR832047**	**OR832067**
*Cytosporafraxinigena*	BBH 42442	*Fraxinusornus*	NA	MF190133	NA	NA	NA	NA
*Cytosporafraxinigena*	MFLUCC 14-0868^T^	*Fraxinusornus*	Italy	MF190133	NA	NA	NA	NA
*Cytosporafugax*	CXY 1371	*Populussimonii*	Jilin, China	KM034852	NA	NA	NA	KM034891
*Cytosporafugax*	CXY 1381	*Populusussuriensis*	Heilongjiang, China	KM034853	NA	NA	NA	KM034890
*Cytosporagalegicola*	MFLUCC 18-1199^T^	*Galegaofficinalis*	Forlì-Cesena, Italy	MK912128	MN685810	MN685820	NA	NA
*Cytosporagigalocus*	CFCC 89620^T^	*Juglansregia*	Qinghai, China	KR045628	KU710997	KU710957	KU710920	KR045669
*Cytosporagigalocus*	CFCC 89621	*Juglansregia*	Qinghai, China	KR045629	KU710998	KU710958	KU710921	KR045670
*Cytosporagigaspora*	CFCC 50014	*Juniperusprocumbens*	Shanxi, China	KR045630	KU710999.	KU710959	KU710922	KR045671
*Cytosporagigaspora*	CFCC 89634^T^	*Salixpsammophila*	Shaanxi, China	KF765671	KU711000	KU710960	KU710923	KR045672
*Cytosporaglobosa*	MFLU 16-2054^T^	*Abiesalba*	Italy	MT177935	NA	MT432212	MT454016	NA
*Cytosporagranati*	CBS 144237^T^	*Punicagranatum*	California, USA	MG971799	MG971949	NA	MG971514	MG971664
*Cytosporahaidianensis*	CFCC 54056	*Euonymusalatus*	Beijing, China	MT360041	MT363978	MT363987	MT363997	MT364007
*Cytosporahaidianensis*	CFCC 54057^T^	*Euonymusalatus*	Beijing, China	MT360042	MT363979	MT363988	MT363998	MT364008
*Cytosporahaidianensis*	CFCC 54184	*Euonymusalatus*	Beijing, China	MT360043	MT363980	MT363989	MT363999	MT364009
***Cytosporahaidianensis***	**CFCC 59450**	***Euonymusjaponicus***	**Beijing, China**	**OR826171**	**OR832004**	**OR832026**	**OR832048**	**OR832068**
***Cytosporahaidianensis***	**CFCC 59475**	***Malus* ‘*American***’	**Beijing, China**	**OR826172**	**OR832005**	**OR832027**	**OR832049**	**OR832069**
***Cytosporahaidianensis***	**CFCC 59471**	**Acerpictumsubsp.mono**	**Beijing, China**	**OR826173**	**OR832006**	**OR832028**	**OR832050**	**OR832070**
***Cytosporahaidianensis***	**CFCC 59536**	**Acerpictumsubsp.mono**	**Beijing, China**	**OR826174**	**OR832007**	**OR832029**	**OR832051**	**OR832071**
*Cytosporahippophaës*	CFCC 89639	*Hippophaërhamnoides*	Gansu, China	KR045632	KU711001	KU710961	KU710924	KR045673
*Cytosporahippophaës*	CFCC 89640	*Hippophaërhamnoides*	Gansu, China	KF765682	KF765730	KU710962	KP310865	KR045674
*Cytosporahuairouensis*	CFCC 56940	*Prunusarmeniaca*	Beijing, China	ON188758	OR662079	OR662096	OR662113	OR662060
*Cytosporahuairouensis*	CFCC 56973	*Prunusarmeniaca*	Beijing, China	ON188759	OR662080	OR662097	OR662114	OR662061
*Cytosporahuairouensis*	CFCC 57286	*Prunusarmeniaca*	Beijing, China	ON188760	OR662081	OR662098	OR662115	OR662062
*Cytosporairanica*	IRAN 4200C^T^	*Malusdomestica*	Arak, Iran	MW295652	MZ014512	MW824359	MW394146	NA
*Cytosporairanica*	IRAN 4628C	*Malusdomestica*	Nahavand, Iran	OM368651	NA	NA	OM372513	NA
*Cytosporajaponica*	CFCC 89956	*Prunuscerasifera*	Ningxia, China	KR045624	KU710993	KU710953	KU710916	KR045665
*Cytosporajaponica*	CFCC 89960	*Prunuscerasifera*	Ningxia, China	KR045625	KU710994	KU710954	KU710917	KR045666
*Cytosporajoaquinensis*	CBS 144235	*Populusdeltoides*	California, USA	MG971895	MG972044	NA	MG971605	MG971761
*Cytosporajunipericola*	BBH 42444	*Juniperuscommunis*	Italy	MF190126	NA	NA	MF377579	NA
*Cytosporajunipericola*	MFLU 17-0882^T^	*Juniperuscommunis*	Italy	MF190125	NA	NA	MF377580	NA
*Cytosporajuniperina*	CFCC 50501^T^	*Juniperusprzewalskii*	Sichuan, China	MH933632	MH933539	MH933602	MH933507	NA
*Cytosporajuniperina*	CFCC 50502	*Juniperusprzewalskii*	Sichuan, China	MH933633	MH933540	MH933603	MH933508	MH933572
*Cytosporajuniperina*	CFCC 50503	*Juniperusprzewalskii*	Sichuan, China	MH933634	MH933541	MH933604	MH933509	NA
*Cytosporakantschavelii*	CXY 1383	*Populusmaximowiczii*	Jilin, China	KM034867	NA	NA	NA	NA
*Cytosporakantschavelii*	CXY 1386	*Populusmaximowiczii*	Chongqing, China	KM034867	NA	NA	NA	NA
*Cytosporakuanchengensis*	CFCC 52464^T^	*Castaneamollissima*	Hebei, China	MK432616	MK442940	MK578076	NA	NA
*Cytosporakuanchengensis*	CFCC 52465	*Castaneamollissima*	Hebei, China	MK432617	MK442941	MK578077	NA	NA
*Cytosporalongispora*	CBS 144236^T^	*Prunusdomestica*	California, USA	MG971905	MG972054	NA	MG971615	MG971764
*Cytosporalongistiolata*	MFLUCC 16-0628	Salix×fragilis	Russia	KY417734	KY417700	KY417802	NA	NA
*Cytosporaleucosperma*	CFCC 89622	*Pyrusbretschneideri*	Gansu, China	KR045616	KU710988	KU710944	KU710911	KR045657
*Cytosporaleucosperma*	CFCC 89894	*Pyrusbretschneideri*	Qinghai, China	KR045617	KU710989	KU710945	KU710912	KR045658
*Cytosporaleucostoma*	CFCC 50023	*Cornusalba*	Shanxi, China	KR045635	KU711003	KU710964	KU710926	KR045676
*Cytosporaleucostoma*	CFCC 50024	*Prunuspseudocerasus*	Qinghai, China	MH933640	MH933547	MH933605	NA	MH933576
*Cytosporaleucostoma*	CFCC 53140	*Prunussibirica*	Beijing, China	MN854445	MN850760	MN850746	MN850753	MN861115
*Cytosporaleucostoma*	CFCC 53141	*Prunussibirica*	Beijing, China	MN854446	MN850761	MN850747	MN850754	MN861116
*Cytosporaleucostoma*	CFCC 53156	*Juglansmandshurica*	Beijing, China	MN854447	MN850762	MN850748	MN850755	MN861117
*Cytosporaleucostoma*	CFCC 53167	*Prunusarmeniaca*	Xinjiang, China	MK673056	MK673026	MK672998	MK672946	MK672972
*Cytosporaleucostoma*	CFCC 53169	*Prunuspersica*	Beijing, China	MK673080	MK673050	MK673020	MK672966	MK672996
*Cytosporaleucostoma*	CFCC 53170	*Prunuspersica*	Beijing, China	MK673081	MK673051	MK673021	MK672967	MK672997
*Cytosporaleucostoma*	CFCC 54680	*Corylusheterophylla*	Beijing, China	MW839857	MW815941	MW815955	MW815890	MW883973
*Cytosporaleucostoma*	CFCC 54681	*Corylusheterophylla*	Beijing, China	MW839857	MW815942	MW815956	MW815891	MW883974
*Cytosporaleucostoma*	CFCC 54682	*Corylusheterophylla*	Beijing, China	MW839857	MW815943	MW815957	MW815892	MW883975
*Cytosporaleucostoma*	CFCC 54683	*Corylusheterophylla*	Beijing, China	MW839857	MW815944	MW815958	MW815893	MW883976
*Cytosporalumnitzericola*	MFLUCC 17-0508^T^	*Lumnitzeraracernosa*	Tailand	MG975778	MH253457	MH253453	NA	NA
*Cytosporamacropycnidia*	CBS 149338	*Vitisvinifera*	USA	OP038094	OP003977	OP095265	OP106954	OP079909
*Cytosporamali*	CFCC 50028	*Maluspumila*	Gansu, China	MH933641	MH933548	MH933606	MH933513	MH933577
*Cytosporamali*	CFCC 50029	*Maluspumila*	Ningxia, China	MH933642	MH933549	MH933607	MH933514	MH933578
*Cytosporamali*	CFCC 50030	*Maluspumila*	Shaanxi, China	MH933643	MH933550	MH933608	MH933524	MH933579
*Cytosporamali*	CFCC 50031	*Crataegus* sp.	Shanxi, China	KR045636	KU711004	KU710965	KU710927	KR045677
*Cytosporamali*	CFCC 50044	*Malusbaccata*	Qinghai, China	KR045637	KU711005	KU710966	KU710928	KR045678
*Cytosporamali-spectabilis*	CFCC 53181^T^	*Malusspectabilis* ‘Royalty’	Xinjiang, China	MK673066	MK673036	MK673006	MK672953	MK672982
*Cytosporamelnikii*	CFCC 89984	*Rhustyphina*	Xinjiang, China	MH933678	MH933551	MH933609	MH933515	MH933580
*Cytosporamelnikii*	MFLUCC 15-0851	*Malusdomestica*	Russia	KY417735	KY417701	KY417803	NA	NA
*Cytosporamelnikii*	MFLUCC 16-0635	Populusnigravar.italica	Russia	KY417736	KY417702	KY417804	NA	NA
*Cytosporamyrtagena*	CFCC 52454	*Castaneamollissima*	Shaanxi, China	MK432614	MK442938	MK578074	NA	NA
*Cytosporamyrtagena*	CFCC 52455	*Castaneamollissima*	Shaanxi, China	MK432615	MK442939	MK578075	NA	NA
*Cytosporanivea*	MFLUCC 15-0860	*Salixacutifolia*	Russia	KY417737	KY417703	KY417805	NA	NA
*Cytosporanivea*	CFCC 89641	*Elaeagnusangustifolia*	Ningxia, China	KF765683	KU711006	KU710967	KU710929	KR045679
*Cytosporanivea*	CFCC 89643	*Salixpsammophila*	Shaanxi, China	KF765685	NA	KU710968	KP310863	KP310829
*Cytosporanotastroma*	NE_TFR5	*Populustremuloides*	USA	JX438632	NA	NA	JX438543	NA
*Cytosporanotastroma*	NE_TFR8	*Populustremuloides*	USA	JX438633	NA	NA	JX438542	NA
*Cytosporaochracea*	CFCC 53164^T^	*Cotoneaster* sp.	Xinjiang, China	MK673060	MK673030	MK673001	MK672949	MK672976
*Cytosporaoleicola*	CBS 144248^T^	*Oleaeuropaea*	California, USA	MG971944	MG972098	NA	MG971660	MG971752
*Cytosporaolivacea*	CFCC 53174	*Prunuscerasifera*	Xinjiang, China	MK673058	MK673028	MK672999	NA	MK672974
*Cytosporaolivacea*	CFCC 53175	*Prunusdulcis*	Xinjiang, China	MK673062	MK673032	MK673003	NA	MK672978
*Cytosporaolivacea*	CFCC 53176^T^	*Sorbustianschanica*	Xinjiang, China	MK673068	MK673038	MK673008	MK672955	MK672984
*Cytosporaolivacea*	CFCC 53177	*Prunusvirginiana*	Xinjiang, China	MK673071	MK673041	MK673011	NA	MK672987
*C.olivarum*	UCD634-Oe CBS 145585	*Oleaeuropaea*	Ventura Co., CA, U.S.A.	MK514094	MK509025	NA	MK509030	MK509035
*C.olivarum*	UCD644-Oe	*Oleaeuropaea*	Ventura Co., CA, U.S.A.	MK514095	MK509026	NA	MK509031	MK509036
*Cytosporapalm*	CXY 1276	*Cotinuscoggygria*	Beijing, China	JN402990	NA	NA	KJ781296	NA
*Cytosporapalm*	CXY 1280^T^	*Cotinuscoggygria*	Beijing, China	JN411939	NA	NA	KJ781297	NA
*Cytosporaparacinnamomea*	CFCC 55453^T^	*Salixmatsudana*	Gansu, China	MZ702594	OK303456	OK303515	OK303576	OK303643
*Cytosporaparacinnamomea*	CFCC 55455^T^	*Salixmatsudana*	Gansu, China	MZ702598	OK303460	OK303519	OK303580	OK303647
*Cytosporaparakantschavelii*	MFLUCC 15-0857^T^	Populus×sibirica	Russia	KY417738	KY417704	KY417806	NA	NA
*Cytosporaparakantschavelii*	MFLUCC 16-0575	*Pyruspyraster*	Russia	KY417739	KY417705	KY417807	NA	NA
*Cytosporaparapistaciae*	CBS 144506^T^	*Pistaciavera*	California, USA	MG971804	MG971954	NA	MG971519	MG971669
*Cytosporaparasitica*	MFLUCC 15-0507^T^	*Malusdomestica*	Russia	KY417740	KY417706	KY417808	NA	NA
*Cytosporaparasitica*	XJAU 2542-1	*Malus* sp.	Xinjiang, China	MH798884	NA	NA	MH813452	NA
*Cytosporaparasitica*	CFCC 53171	*Maluspumila*	Xinjiang, China	MK673061	MK673031	MK673002	MK672950	MK672977
*Cytosporaparasitica*	CFCC 53172	*Maluspumila*	Xinjiang, China	MK673069	MK673039	MK673009	MK672956	MK672985
*Cytosporaparasitica*	CFCC 53173	*Berberis* sp.	Xinjiang, China	MK673070	MK673040	MK673010	MK672957	MK672986
*Cytosporaparatranslucens*	MFLUCC 15-0506^T^	Populusalbavar.bolleana	Russia	KY417741	KY417707	KY417809	NA	NA
*Cytosporaparatranslucens*	MFLUCC 16-0627	*Populusalba*	Russia	KY417742	KY417708	KY417810	NA	NA
*Cytosporaparaplurivora*	FDS-439	*Prunusarmeniaca*	Canada	OL640182	OL631586	NA	OL631589	NA
*Cytosporaparaplurivora*	FDS-564	Prunuspersicavar.nucipersica	Canada	OL640183	OL631587	NA	OL631590	NA
*Cytosporaparaplurivora*	FDS-623	Prunuspersicavar.persica	Canada	OL640181	OL631588	NA	OL631591	NA
*Cytosporaphialidica*	MFLUCC 17-2498	*Alnusglutinosa*	Italy	MT177932	NA	MT432209	MT454014	NA
*Cytosporapiceae*	CFCC 52841^T^	*Piceacrassifolia*	Xinjiang, China	MH820398	MH820406	MH820395	MH820402	MH820387
*Cytosporapiceae*	CFCC 52842	*Piceacrassifolia*	Xinjiang, China	MH820399	MH820407	MH820396	MH820403	MH820388
***Cytosporapinea***	**CFCC 59521^T^**	***Pinusbungeanae***	**Beijing, China**	**OR826181**	**OR832014**	**OR832036**	**OR832058**	**OR832078**
***Cytosporapinea***	**CFCC 59522**	***Pinusbungeanae***	**Beijing, China**	**OR826182**	**OR832015**	**OR832037**	**OR832059**	**OR832079**
***Cytosporapinea***	**CFCC 59523**	***Pinusbungeanae***	**Beijing, China**	**OR826183**	**OR832016**	**OR832038**	**OR832060**	**OR832080**
***Cytosporapinea***	**CFCC 59524**	***Pinusbungeanae***	**Beijing, China**	**OR826184**	**OR832017**	**OR832039**	**OR832061**	**OR832081**
*Cytosporapingbianensis*	MFLUCC 18-1204^T^	Undefined wood	Yunnan, China	MK912135	MN685817	MN685826	NA	NA
*Cytosporapistaciae*	CBS 144238^T^	*Pistaciavera*	California, USA	MG971802	MG971952	NA	MG971517	MG971667
*Cytosporaplatanicola*	MFLU 17-0327	*Platanushybrida*	Italy	MH253451	MH253449	MH253450	NA	NA
*Cytosporaplatyclada*	CFCC 50504^T^	*Platycladusorientalis*	Yunnan, China	MH933645	MH933552	MH933610	MH933516	MH933581
*Cytosporaplatyclada*	CFCC 50505	*Platycladusorientalis*	Yunnan, China	MH933646	MH933553	MH933611	MH933517	MH933582
*Cytosporaplatyclada*	CFCC 50506	*Platycladusorientalis*	Yunnan, China	MH933647	MH933554	MH933612	MH933518	MH933583
*Cytosporaplatycladicola*	CFCC 50038^T^	*Platycladusorientalis*	Gansu, China	KT222840	MH933555	MH933613	MH933519	MH933584
*Cytosporaplatycladicola*	CFCC 50039	*Platycladusorientalis*	Gansu, China	KR045642	KU711008	KU710973	KU710931	KR045683
*Cytosporaplurivora*	CBS 144239^T^	*Oleaeuropaea*	California, USA	MG971861	MG972010	NA	MG971572	MG971726
*Cytosporapopulicola*	CBS 144240	*Populusdeltoides*	California, USA	MG971891	MG972040	NA	MG971601	MG971757
*Cytosporapopulina*	CFCC 89644^T^	*Salixpsammophila*	Shaanxi, China	KF765686	KU711007	KU710969	KU710930	KR045681
*Cytosporapopulinopsis*	CFCC 50032^T^	*Sorbusaucuparia*	Ningxia, China	MH933648	MH933556	MH933614	MH933520	MH933585
*Cytosporapopulinopsis*	CFCC 50033	*Sorbusaucuparia*	Ningxia, China	MH933649	MH933557	MH933615	MH933521	MH933586
*Cytosporapredappioensis*	MFLUCC 17-2458^T^	*Platanushybrida*	Italy	MG873484	NA	NA	NA	NA
*Cytosporaprunicola*	MFLU 17-0995^T^	*Prunus* sp.	Italy	MG742350	MG742353	MG742352	NA	NA
*Cytosporapruni-mume*	CFCC 53179	*Prunusarmeniaca*	Xinjiang, China	MK673057	MK673027	NA	MK672947	MK672973
*Cytosporapruni-mume*	CFCC 53180^T^	*Prunusmume*	Xinjiang, China	MK673067	MK673037	MK673007	MK672954	MK672983
*Cytosporaprunina*	CFCC 58997	*Prunusarmeniaca*	Beijing, China	OR578808	NA	NA	NA	OR662077
*Cytosporaprunina*	CFCC 58998	*Prunusarmeniaca*	Beijing, China	OR578809	NA	NA	NA	OR662078
*Cytosporapruinopsis*	CFCC 50034^T^	*Ulmuspumila*	Shaanxi, China	KP281259	KP310836	KU710970	KP310849	KP310819
*Cytosporapruinopsis*	CFCC 50035	*Ulmuspumila*	Jilin, China	KP281260	KP310837	KU710971	KP310850	KP310820
*Cytosporapruinopsis*	CFCC 53153	*Ulmuspumila*	Beijing, China	MN854451	MN850763	MN850752	MN850759	MN861121
*Cytosporapruinosa*	CFCC 50036	*Syringaoblata*	Qinghai, China	KP310800	KP310832	NA	KP310845	KP310815
*Cytosporapruinosa*	CFCC 50037	*Syringaoblata*	Qinghai, China	MH933650	MH933558	NA	MH933522	MH933589
*Cytosporapubescentis*	MFLUCC 18-1201^T^	*Quercuspubescens*	Forlì-Cesena, Italy	MK912130	MN685812	MN685821	NA	NA
*Cytosporapunicae*	CBS 144244	*Punicagranatum*	California, USA	MG971943	MG972091	NA	MG971654	MG971798
*Cytosporaquercicola*	MFLU 17-0881	*Quercus* sp.	Italy	MF190128	NA	NA	NA	NA
*Cytosporaquercicola*	MFLUCC 14-0867^T^	*Quercus* sp.	Italy	MF190129	NA	NA	NA	NA
*Cytosporaribis*	CFCC 50026	*Ulmuspumila*	Qinghai, China	KP281267	KP310843	KU710972	KP310856	KP310826
*Cytosporaribis*	CFCC 50027	*Ulmuspumila*	Qinghai, China	KP281268	KP310844	NA	KP310857	KP310827
*Cytosporarosae*	MFLU 17-0885	*Rosacanina*	Italy	MF190131	NA	NA	NA	NA
*Cytosporarosicola*	CF 20197024^T^	*Rosa* sp.	Tibet, China	MK673079	MK673049	MK673019	MK672965	MK672995
*Cytosporarosigena*	MFLUCC 18-0921^T^	*Rosa* sp.	Russia	MN879872	NA	NA	NA	NA
*Cytosporarostrata*	CFCC 89909	*Salixcupularis*	Gansu, China	KR045643	KU711009	KU710974	KU710932	KR045684
*Cytosporarostrata*	CFCC 89910	*Salixcupularis*	Gansu, China	KR045644	KU711010	KU710975	KU710933	NA
*Cytosporarusanovii*	MFLUCC 15-0853	Populus×sibirica	Russia	KY417743	KY417709	KY417811	NA	NA
*Cytosporarusanovii*	MFLUCC 15-0854^T^	*Salixbabylonica*	Russia	KY417744	KY417710	KY417812	NA	NA
*Cytosporasalicacearum*	MFLUCC 15-0509	*Salixalba*	Russia	KY417746	KY417712	KY417814	NA	NA
*Cytosporasalicacearum*	MFLUCC 15-0861	Salix×fragilis	Russia	KY417745	KY417711	KY417813	NA	NA
*Cytosporasalicacearum*	MFLUCC 16-0587	*Prunuscerasus*	Russia	KY417742	KY417708	KY417810	NA	NA
*Cytosporasalicacearum*	MFLUCC 16-0576	Populusnigravar.italica	Russia	KY417741	KY417707	KY417809	NA	NA
*Cytosporasalicicola*	MFLUCC 14-1052^T^	*Salixalba*	Russia	KU982636	KU982637	NA	NA	NA
*Cytosporasalicicola*	MFLUCC 15-0866	*Salix* sp.	Thailand	KY417749	KY417715	KY417817	NA	NA
*Cytosporasalicina*	MFLUCC 15-0862	*Salixalba*	Russia	KY417750	KY417716	KY417818	NA	NA
*Cytosporasalicina*	MFLUCC 16-0637	Salix×fragilis	Russia	KY417751	KY417717	KY417819	NA	NA
*Cytosporaschulzeri*	CFCC 50042	*Maluspumila*	Gansu, China	KR045650	KU711014	KU710981	KU710937	KR045691
*Cytosporasibiraeae*	CFCC 50045^T^	*Sibiraeaangustata*	Gansu, China	KR045651	KU711015	KU710982	KU710938	KR045692
*Cytosporasibiraeae*	CFCC 50046	*Sibiraeaangustata*	Gansu, China	KR045652	KU711015	KU710983	KU710939	KR045693
*Cytosporasophorae*	CFCC 50047	*Styphnolobiumjaponicum*	Shanxi, China	KR045653	KU711017	KU710984	KU710940	KR045694
*Cytosporasophorae*	CFCC 50048	*Magnoliagrandiflora*	Shanxi, China	MH820401	MH820409	MH820397	MH820405	MH820390
*Cytosporasophorae*	CFCC 89598	*Styphnolobiumjaponicum*	Gansu, China	KR045654	KU711018	KU710985	KU710941	KR045695
*Cytosporasophoricola*	CFCC 89596	*Styphnolobiumjaponicumvar.pendula*	Gansu, China	KR045656	KU711020	KU710987	KU710943	KR045697
*Cytosporasophoricola*	CFCC 89595^T^	*Styphnolobiumjaponicumvar.pendula*	Gansu, China	KR045655	KU711019	KU710986	KU710942	KR045696
*Cytosporasophoriopsis*	CFCC 55469	*Salixmatsudana*	Gansu, China	MZ702583	OK303445	OK303504	OK303565	OK303632
*Cytosporasophoriopsis*	CFCC 89600	*Styphnolobiumjaponicum*	Gansu, China	KR045623	KU710992	KU710951	KU710915	KP310817
***Cytosporasorbariae***	**CFCC 59443**	***Sorbariasorbifolia***	**Beijing, China**	**OR826175**	**OR832008**	**OR832030**	**OR832052**	**OR832072**
***Cytosporasorbariae***	**CFCC 59445^T^**	***Sorbariasorbifolia***	**Beijing, China**	**OR826176**	**OR832009**	**OR832031**	**OR832053**	**OR832073**
***Cytosporasorbariae***	**CFCC 59529**	***Sorbariasorbifolia***	**Beijing, China**	**OR826177**	**OR832010**	**OR832032**	**OR832054**	**OR832074**
***Cytosporasorbariae***	**CFCC 59530**	***Sorbariasorbifolia***	**Beijing, China**	**OR826178**	**OR832011**	**OR832033**	**OR832055**	**OR832075**
*Cytosporasorbi*	MFLUCC 16-0631^T^	*Sorbusaucuparia*	Russia	KY417752	KY417718	KY417820	NA	NA
*Cytosporasorbicola*	MFLUCC 16-0584^T^	*Acerpseudoplatanus*	Russia	KY417755	KY417721	KY417823	NA	NA
*Cytosporasorbicola*	MFLUCC 16-0633	*Cotoneastermelanocarpus*	Russia	KY417758	KY417724	KY417826	NA	NA
*Cytosporasorbina*	CF 20197660^T^	*Sorbustianschanica*	Xinjiang, China	MK673052	MK673022	NA	MK672943	MK672968
*Cytosporaspiraeae*	CFCC 50049^T^	*Spiraeasalicifolia*	Gansu, China	MG707859	MG708196	MG708199	NA	NA
*Cytosporaspiraeae*	CFCC 50050	*Spiraeasalicifolia*	Gansu, China	MG707860	MG708197	MG708200	NA	NA
*Cytosporaspiraeicola*	CFCC 53138^T^	*Spiraeasalicifolia*	Beijing, China	MN854448	NA	MN850749	MN850756	MN861118
*Cytosporaspiraeicola*	CFCC 53139	*Tilianobilis*	Beijing, China	MN854449	NA	MN850750	MN850757	MN861119
*Cytosporatamaricicola*	CFCC 50507	*Rosamultifolora*	Yunnan, China	MH933651	MH933559	MH933616	MH933525	MH933587
*Cytosporatamaricicola*	CFCC 50508^T^	*Tamarixchinensis*	Yunnan, China	MH933652	MH933560	MH933617	MH933523	MH933588
*Cytosporatanaitica*	MFLUCC 14-1057^T^	*Betulapubescens*	Russia	KT459411	KT459413	NA	NA	NA
*Cytosporathailandica*	MFLUCC 17-0262^T^	*Xylocarpusmoluccensis*	Thailand	MG975776	MH253459	MH253455	NA	NA
*Cytosporathailandica*	MFLUCC 17-0263^T^	*Xylocarpusmoluccensis*	Thailand	MG975777	MH253460	MH253456	NA	NA
*Cytosporatibetensis*	CF 20197026	*Cotoneaster* sp.	Tibet, China	MK673076	MK673046	MK673016	MK672962	MK672992
*Cytosporatibetensis*	CF 20197029	*Cotoneaster* sp.	Tibet, China	MK673077	MK673047	MK673017	MK672963	MK672993
*Cytosporatibetensis*	CF 20197032^T^	*Cotoneaster* sp.	Tibet, China	MK673078	MK673048	MK673018	MK672964	MK672994
*Cytosporatibouchinae*	CPC 26333^T^	*Tibouchinasemidecandra*	France	KX228284	NA	NA	NA	NA
*Cytosporatranslucens*	CXY 1351	*Populusdavidiana*	Inner Mongolia, China	KM034874	NA	NA	NA	KM034895
*Cytosporatranslucens*	CXY 1359	*Populus* × *Beijingensis*	Beijing, China	KM034871	NA	NA	NA	KM034894
*Cytosporaulmi*	MFLUCC 15-0863^T^	*Ulmusminor*	Russia	KY417759	NA	NA	NA	NA
*Cytosporaverrucosa*	CFCC 53157 ^T^	*Platycladusorientalis*	Beijing, China	MW418408	NA	MW422911	MW422923	MW422935
*Cytosporaverrucosa*	CFCC 53158	*Platycladusorientalis*	Beijing, China	MW418410	MW422901	MW422913	MW422925	MW422937
*Cytosporaverrucosa*	CFCC 54369	*Platycladusorientalis*	Beijing, China	MW418409	NA	MW422912	MW422924	MW422936
*Cytosporaverrucosa*	CFCC 54370	*Platycladusorientalis*	Beijing, China	MW418411	MW422902	MW422914	MW422926	MW422938
*Cytosporavinacea*	CBS 141585^T^	*Vitisinterspecific* hybrid ‘Vidal’	USA	KX256256	NA	NA	KX256277	KX256235
*Cytosporaviridistroma*	CBS 202.36^T^	*Cerciscanadensis*	USA	MN172408	NA	NA	MN271853	NA
*Cytosporaviticola*	Cyt2	*Vitisinterspecific* hybrid ‘Frontenac’	USA	KX256238	NA	NA	KX256259	KX256217
*Cytosporaviticola*	CBS 141586^T^	*Vitisvinifera* ‘CabernetFranc’	USA	KX256239	NA	NA	KX256260	KX256218
*Cytosporaxinjiangensis*	CFCC 53182	*Rosa* sp.	Xinjiang, China	MK673064	MK673034	MK673004	MK672951	MK672980
*Cytosporaxinjiangensis*	CFCC 53183^T^	*Rosa* sp.	Xinjiang, China	MK673065	MK673035	MK673005	MK672952	MK672981
*Cytosporaxinglongensis*	CFCC 52458^T^	*Castaneamollissima*	Hebei, China	MK432622	MK442946	MK578082	NA	NA
*Cytosporaxinglongensis*	CFCC 52459	*Castaneamollissima*	Hebei, China	MK432623	MK442947	MK578083	NA	NA
*Cytosporaxylocarpi*	MFLUCC 17-0251^T^	*Xylocarpusgranatum*	Thailand	MG975775	MH253458	MH253454	NA	NA
*Cytosporayakimana*	CBS 149297	*Vitisvinifera*	USA	OM976602	ON012555	ON045093	ON012569	ON086750
*Cytosporayakimana*	CBS 149298	*Vitisvinifera*	USA	OM976603	ON012556	ON045094	ON012570	ON086751
*Cytosporazhaitangensis*	CFCC 56227^T^	*Euonymusjaponicus*	Beijing, China	OQ344750	OQ398760	OQ398789	OQ410623	OQ398733
*Cytosporazhaitangensis*	CFCC 57537	*Euonymusjaponicus*	Beijing, China	OQ344751	OQ398761	OQ398790	OQ410624	OQ398734
*Diaporthevaccinii*	CBS 160.32	*Vacciniummacrocarpon*	USA	KC343228	JQ807297	NA	KC343954	KC344196

^1^Acronyms: ATCC: American Type Culture Collection, Virginia, USA; BBH: BIOTEC Bangkok Herbarium, National Science and Technology Development Agency, Thailand; CBS: Westerdijk Fungal Biodiversity Institute (CBS-KNAW Fungal Biodiversity Centre), Utrecht, The Netherlands; CFCC: China Forestry Culture Collection Centre, Beijing, China; CMW: Culture Collection of Michael Wingfield, University of Pretoria, South Africa; CPC: Culture Collection of Pedro Crous, The Netherlands; IMI: Culture Collection of the International Mycological Institute, CABI Bioscience, Egham, Surrey, UK; MFLU: Mae Fah Luang University herbarium, Thailand; MFLUCC: Mae Fah Luang University Culture Collection, Thailand; MUCC: Murdoch University Culture Collection, Perth, Australia; NE: Gerard Adams Collections, University of Nebraska, Lincoln NE; PPRI: Culture Collection of the Plant Protection Research Institute, Agriculture Research Center, Pretoria, South Africa; XJAU: Xinjiang Agricultural University, Xinjiang, China; NA: not applicable. All the new isolates used in this study are in bold and the type materials are marked with T.

## ﻿Results

### ﻿Phylogenetic analyses

**Figure 2.**
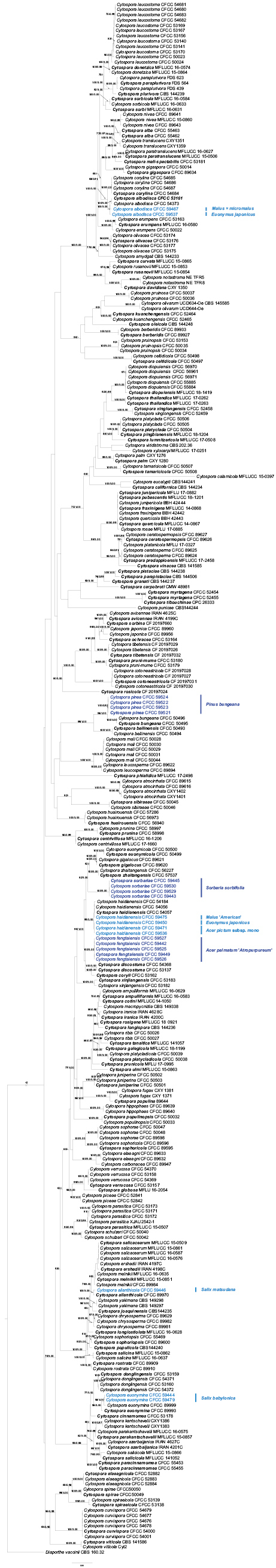
Phylogram of *Cytospora* based on Maximum Likelihood (ML) analysis of the dataset of combined ITS, *act*, *rpb2*, *tef1-a* and *tub2* genes. Numbers above the branches indicate ML bootstrap values (ML-BS ≥ 60%) and Bayesian Posterior Probabilities (BPP ≥ 0.9). Ex-type isolates are in bold. lsolates in this study marked with its hosts and highlighted in two different colours where the novel species are shown in dark blue and the known species are shown in light blue.

Each gene region and the combined matrix of five gene sequences of *Cytospora* were both considered. The concatenated alignment comprised sequences from 296 strains and *Diaporthevaccinii*CBS 160.32 was selected as the outgroup. *Cytospora* ingroup strains with a total of 3166 characters including gaps (615 characters for ITS, 344 for *act*, 731 for *rpb2*, 811 for *tef1-α* and 665 for *tub2*). ML bootstraps (ML BS ≥ 60%) and Bayesian posterior probabilities (BPP ≥ 0.90) have been shown above the branches (Fig. 2). For ML analysis, the substitution model (GTR+G+I model) for each dataset was selected following recent studies (Fan et al. 2020; Pan et al. 2020, 2021). Confidence levels for the nodes were determined using 1,000 replicates of bootstrapping methods (Hillis and Bull 1993). The matrix had 1992 distinct alignment patterns. Estimated base frequencies are as follows: A = 0.244402, C = 0.286560, G = 0.238889, T = 0.230150; substitution rates: AC = 1.282426, AG = 3.546575, AT = 1.431177, CG = 0.946427, CT = 6.172877, GT = 1.000000; gamma distribution shape parameter: α = 0.364165. For BI analysis, the best-fit model of nucleotide evolution was deduced on the AIC (ITS and *act*: GTR+I+G; *rpb2* and *tef1-α*: TrN+I+G; and *tub2*: HKY+I+G).

The topologies resulting from ML and BI analyses of the concatenated dataset were similar. In the present study, 22 isolates formed seven clades representing seven species, of which four clades were grouped with the strains of four known species (*C.ailanthicola*, *C.albodisca*, *C.euonymina*, *C.haidianensis*). Isolates in other three clades were separated from all other species and were also highly supported (ML/BI = 100/1) (Fig. 2), representing three new species (*C.fengtaiensis*, *C.pinea*, *C.sorbariae*), which have been described below.

### ﻿Taxonomy

#### ﻿ Cytospora ailanthicola

Taxon classificationFungiDiaporthalesValsaceae

X.L. Fan & C.M. Tian, Persoonia 45: 13 (2020)

5E4BEF0C-DDA0-5FFC-8E9B-8661BDC18B68

Fig. 3

##### Description.

***Sexual morph***: not observed. ***Asexual morph*: *Conidiomata pycnidial***, immersed in the bark, scattered, producing black area on bark, circular to ovoid, with multiple locules, occasionally slightly erumpent through the surface. ***Conceptacle*** absent. ***Ectostromatic disc*** inconspicuous, grey to black, circular to ovoid, producing one ostiole per disc when mature. ***Ostiole*** in the centre of the disc, black, 50–110 µm in diam. ***Locules*** numerous, subdivided frequently by invaginations with common walls, circular to ovoid, 300–500 µm in diam. ***Conidiophores*** hyaline, unbranched, approximately cylindrical, 6.5–9 × 1–1.5 (av. = 8 ± 1.5 × 1.3 ± 0.2, n = 50) µm. ***Conidiogenous cells*** enteroblastic, phialidic. ***Conidia*** hyaline, elongate-allantoid, smooth, aseptate, 2.8–3 × 0.8–1.2 (av. = 3 ± 0.3 × 1 ± 0.2, n = 50) µm.

**Figure 3.**
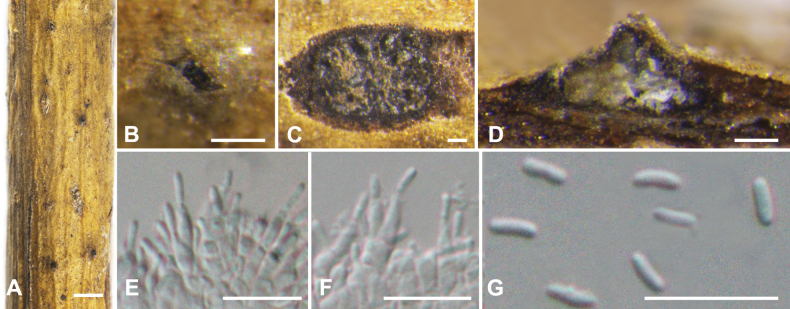
*Cytosporaailanthicola* from *Salixmatsudana* (BJFC CF20230400) **A, B** habit of conidiomata on branch **C** transverse section through conidiomata **D** longitudinal section through conidiomata **E, F** conidiophores and conidiogenous cells **G** conidia. Scale bars: 1 mm (**A**); 200 µm (**B**); 100 µm (**C, D**); 10 µm (**E–G**)

##### Culture characteristics.

Cultures on PDA are initially white, growing fast up to 5 cm after 3 d and entirely covering the 6 cm Petri dish after 7 d, with fluffy and whitish aerial mycelium, producing black pycnidia with cream to yellowish conidial drops exuding from the ostioles after 30 d. *Pycnidia* aggregated on surface.

##### Materials examined.

China, Beijing City, Fengtai Distinct, Qianling Mountain scenic area, 39°51'12.28"N, 116°5'17.74"E, from branches of *Salixmatsudana*, 12 Apr 2023, A.L. Jia & X.L. Fan (BJFC CF20230400, living culture CFCC 59446).

##### Notes.

*Cytosporaailanthicola* was first observed on branches of *Ailanthusaltissima* in China by Fan et al. (2020). Lin et al. (2022) confirmed this species was a pathogen with strong virulence caused by poplar canker disease. In this study, CFCC 59446 was isolated from symptomatic branches of *Salixmatsudana* in Beijing, which clustered in a well-supported clade with *C.ailanthicola* ex-holotype CFCC 89970 (ML/BI = 100/1). Therefore, CFCC 59446 is identified as *C.ailanthicola*.

#### ﻿ Cytospora albodisca

Taxon classificationFungiDiaporthalesValsaceae

M. Pan & X.L. Fan, Front. Plant Sci. 12 (636460): 3 (2021).

F29C59FF-DEAF-5047-B4E8-86EED2D4AFF1

Fig. 4

##### Description.

***Sexual morph***: not observed. ***Asexual morph*: *Conidiomata pycnidial***, semi-immersed in the bark, scattered, producing black area on bark, circular to ovoid, with multiple locules, occasionally slightly erumpent through the surface. ***Conceptacle*** absent. ***Ectostromatic disc*** conspicuous, black, discoid, circular to ovoid, 680–1200 µm in diam., producing one ostiole per disc when mature. ***Ostiole*** grey to black, in the centre of the disc, 140–300 µm in diam. ***Locules*** numerous, subdivided frequently by invaginations with common walls, circular to ovoid, 500–1200 µm in diam. ***Conidiophores*** hyaline, unbranched, approximately cylindrical, 7–11× 0.8–2 (av. = 9 ± 2.2 × 1.3 ± 0.3, n = 50) µm. ***Conidiogenous cells*** enteroblastic, phialidic. ***Conidia*** hyaline, elongate-allantoid, smooth, aseptate, 5–7 × 1–2 (av. = 6 ± 0.5 × 1.5 ± 0.3, n = 50) µm.

##### Culture characteristics.

Cultures on PDA are initially white, growing fast up to 5 cm in diam. after 3 d and entirely covering the 6 cm Petri dish after 5 d, becoming dark herbage green to dull green after 7–10 d. Colonies are sparse in the centre and compact to the margin. After 30 d, *pycnidia* distributed irregularly on surface.

**Figure 4.**
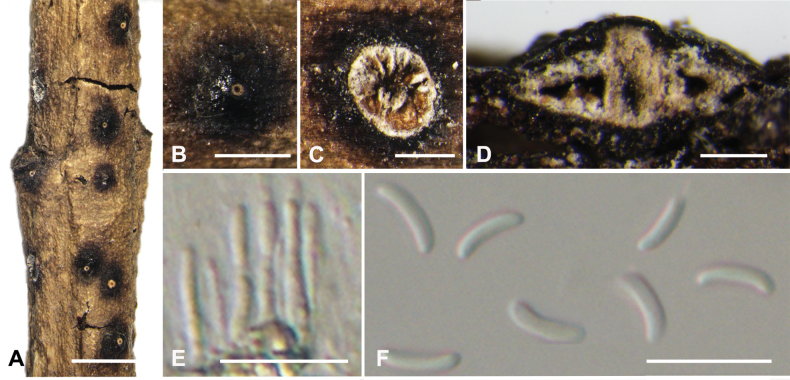
*Cytosporaalbodisca* from *Euonymusjaponicus* (BJFC CF20230402) **A, B** habit of conidiomata on branch **C** transverse section through conidiomata **D** longitudinal section through conidiomata **E** conidiophores and conidiogenous cells **F** conidia. Scale bars: 2 mm (**A**); 1 mm (**B**); 500 µm (**C**); 200 µm (**D**); 10 µm (**E**, F).

##### Materials examined.

China, Beijing City, Fengtai Distinct, Qianling Mountain scenic area, 39°51'12.28"N, 116°5'17.74"E, from branches of Malus×micromalus, 12 Apr 2023, A.L. Jia & X.L. Fan (BJFC CF20230401, living culture CFCC 59467); Qianling Mountain scenic area, 39°51'12.28"N, 116°5'17.74"E, from branches of *Euonymusjaponicus*, 12 Apr 2023, A.L. Jia & X.L. Fan (BJFC CF20230402, living culture CFCC 59537).

##### Notes.

*Cytosporaalbodisca* was described by Pan et al. (2021) associated with canker disease of *Platycladusorientalis* in China. It can be identified by having ascostroma surrounded by a black conceptacle, producing allantoid, aseptate ascospores (8–14 × 2–3.5 µm). In this study, the asexual morph of *Cytosporaalbodisca* is characterised by the pycnidial stromata submerged in the cortex with multiple locules, filamentous conidiophores producing hyaline, allantoid, eguttulate and smooth conidia. Phylogenetically, the isolates (CFCC 59459 and 59537) clustered together with *C.albodisca* with high statistical support (ML/BI = 100/1) (Fig. 2). Therefore, the isolate in this study was confirmed to be *C.albodisca*.

#### ﻿ Cytospora euonymina

Taxon classificationFungiDiaporthalesValsaceae

X.L. Fan & C.M. Tian, Persoonia 45: 21 (2020)

F839798C-D04F-5EB4-A09C-2A896F322F93

Fig. 5

##### Description.

***Sexual morph***: not observed. ***Asexual morph*: *Conidiomata pycnidial***, immersed in the bark, scattered, producing black area on bark, erumpent through the surface, with multiple locules. ***Conceptacle*** absent. ***Ectostromatic disc*** honey to dark mouse grey, conspicuous, circular to ovoid, 200–500µm in diam, with one ostiole per disc. ***Ostiole*** in the centre of the disc, black, conspicuous, 80–200 μm diam. ***Locules*** numerous, subdivided frequently by invaginations with common walls, 400–750 µm in diam. ***Conidiophores*** borne along the locules, hyaline, unbranched or occasionally branched at the base or in the middle, thin-walled, 8–12 × 1.5–2 (av. = 10 ± 2.1 × 1.8 ± 0.3, n = 50) μm, embedded in a gelatinous layer. ***Conidiogenous cells*** enteroblastic, phialidic. ***Conidia*** hyaline, elongate-allantoid, smooth, aseptate, 5–7 × 1–2 (av. = 6 ± 0.5 × 1.5 ± 0.3, n = 50) µm.

##### Culture characteristics.

Cultures on PDA are initially white, irregular, lacking aerial mycelium, fast growing up to 5 cm diam. after 3 d. Colonies pale white to light salmon after 30 d, pycnidia distributed sparsely over the medium surface.

**Figure 5.**
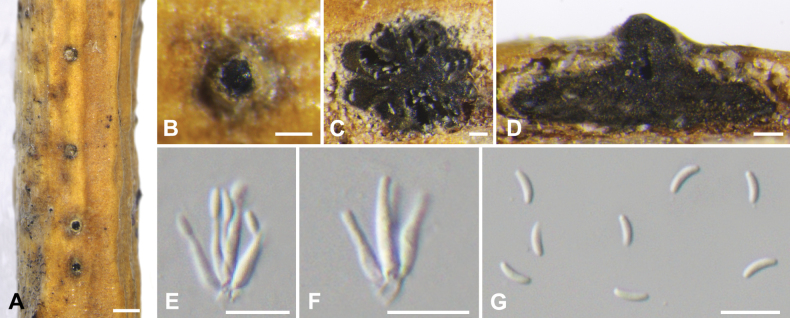
*Cytosporaeuonymina* from *Salixbabylonica* (BJFC CF20230403) **A, B** habit of conidiomata on branch **C** transverse section through conidiomata **D** longitudinal section through conidiomata **E, F** conidiophores and conidiogenous cells **G** conidia. Scale bars: 500 µm (**A**); 200 µm (**B**); 100 µm (**C, D**); 10 µm (**E–G**).

##### Materials examined.

China, Beijing City, Fengtai Distinct, Qianling Mountain scenic area, 39°51'12.28"N, 116°5'17.74"E, from branches of *Salixbabylonica*, 12 Apr 2023, A.L. Jia & X.L. Fan (BJFC CF20230403, living culture CFCC 59444; BJFC CF20230404, living culture CFCC 59479).

##### Notes.

*Cytosporaeuonymina* was isolated from *Euonymuskiautschovicus* in Shanxi Province, China (Fan et al. 2020). It is characterised by having pycnidia covered by the darkened cuticle. Lin et al. (2023b) reported this species from leaves of *Euonymusjaponicus.* In this study, two isolates grouped together with *C.euonymina* in ML and BI trees (ML/BI = 98/1). Therefore, they were identified as *C.euonymina*. Additionally, CFCC 59444 and 59479 extends its host range which were isolated from branches of *Salixbabylonica* in the current study.

#### ﻿ Cytospora fengtaiensis

Taxon classificationFungiDiaporthalesValsaceae

A.L. Jia & X.L. Fan sp. nov.

373B0659-4608-582B-A62E-D1A55886607D

850894

Fig. 6

##### Etymology.

Named after the place where it was first collected, Fengtai District, Beijing City.

##### Typification.

China. Beijing City, Fengtai District, Qianling Mountain scenic area, 39°51'12.28"N, 116°5'17.74"E, from branches of *Acerpalmatum* ‘*Atropurpureum*’, 7 Apr 2023, A.L. Jia & X.L. Fan (holotype BJFC CF20230405, ex-holotype living culture CFCC 59449); 39°51'12.51"N, 116°5'17.32"E, from branches of *Acerpalmatum* ‘*Atropurpureum*’, 7 Apr 2023, A.L. Jia & X.L. Fan (paratype BJFC CF20230406, ex-paratype living culture CFCC 59442.

##### Description.

***Sexual morph***: not observed. ***Asexual morph*: *Conidiomata pycnidial***, immersed in the bark, scattered, producing black area on bark, circular to ovoid, with multiple locules, occasionally slightly erumpent through the surface. ***Conceptacle*** absent. ***Ectostromatic disc*** conspicuous, grey to black, discoid, circular to ovoid, 180–250 µm in diam., producing one ostiole per disc when mature. ***Ostiole*** grey to black, nearly at the same level as the disc surface, 70–105 µm in diam. Locules numerous, subdivided frequently by invaginations with common walls, circular to ovoid, 560–800 µm in diam. ***Conidiophores*** hyaline, unbranched, approximately cylindrical, 11–17 × 1.5–2 (av. = 14.7 ± 2.7 × 1.6 ± 0.3, n = 50) µm. ***Conidiogenous cells*** enteroblastic, phialidic. ***Conidia*** hyaline, elongate-allantoid, smooth, aseptate, 5–6 × 1–2 (av. = 5.5 ± 0.5 × 1.6 ± 0.2, n = 50) µm.

##### Culture characteristics.

Cultures on PDA are initially white to pale vinaceous, growing slowly up to 3 cm after 3 d and entirely covering the 6 cm Petri dish after 7 d, becoming fawn after 14 d. Colonies are flat with a uniform texture, Colony margin irregular. After 30 d, *pycnidia* aggregated on surface.

**Figure 6.**
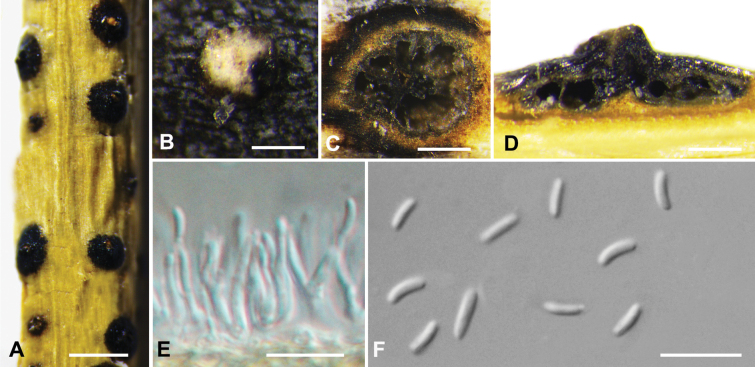
*Cytosporafengtaiensis* from *Acerpalmatum* ‘*Atropurpureum*’ (BJFC CF20230405) **A, B** habit of conidiomata on branch **C** transverse section through conidiomata **D** longitudinal section through conidiomata **E** conidiophores and conidiogenous cells **F** conidia. Scale bars: 1 mm (**A**); 200 µm (**B–D**); 10 µm (**E, F**).

##### Additional materials examined.

China. Beijing City, Fengtai District, Qianling Mountain scenic area, 39°51'11.45"N, 116°5'15.36"E, from branches of *Acerpalmatum* ‘*Atropurpureum*’, 7 Apr 2023, A.L. Jia & X.L. Fan (BJFC CF20230407, living culture CFCC 59525; BJFC CF20230408, living cultures CFCC 59526 and 59527).

##### Notes.

*Cytosporafengtaiensis* is associated with canker disease of *Acerpalmatum* ‘*Atropurpureum*’ in the current study. It can be identified by its conidiomata producing larger black areas on bark. Phylogenetically, five isolates in this study formed a distinct lineage in the phylogenetic trees of each individual gene (ITS, *act*, *rpb2*, *tef1-α* and *tub2*) and the combined gene dataset (Fig. 2).

#### ﻿ Cytospora haidianensis

Taxon classificationFungiDiaporthalesValsaceae

X. Zhou & X.L. Fan, Forests 11: 524 (2020)

494EAF10-355F-5445-8682-D613412A1372

Fig. 7

##### Description.

***Sexual morph***: not observed. ***Asexual morph*: *Conidiomata pycnidial***, immersed in the bark, scattered, producing black area on bark, circular to ovoid, with multiple locules, occasionally slightly erumpent through the surface. ***Conceptacle*** absent. ***Ectostromatic disc*** isabelline to dark brick, conspicuous, circular to ovoid, 130–350 µm in diam, with one ostiole per disc. ***Ostiole*** in the centre of the disc, black, conspicuous, 90–180 µm in diam. ***Locules*** numerous, subdivided frequently by invaginations with common walls, circular to ovoid, 500–1200 µm in diam. ***Conidiophores*** hyaline, branched at the base or unbranched, approximately cylindrical, 12–19 × 1–1.5 (av. = 15.5 ± 4.3 × 1.1 ± 0.4, n = 50) µm. ***Conidiogenous cells*** enteroblastic, phialidic, subcylindrical to cylindrical. ***Conidia*** hyaline, elongate-allantoid, smooth, aseptate, thin-walled, 4.8–6 × 1.5–2 (av. = 5.3 ± 0.7 × 1.7 ± 0.3, n = 50) µm.

##### Cultural characteristics.

Colonies on PDA are initially white after 3 d, becoming light brown after 14 d. The colonies are thin with a uniform texture, lacking aerial mycelium. *Pycnidia* were randomly observed on the surface of the colony after 30 d.

**Figure 7.**
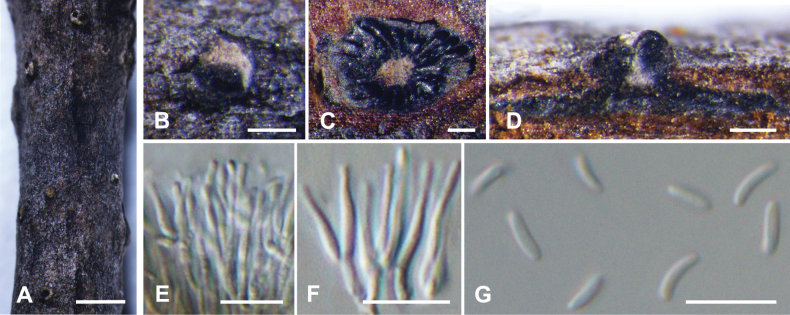
*Cytosporahaidianensis* from *Salixbabylonica* (BJFC CF20230411) **A, B** habit of conidiomata on branch **C** transverse section through conidiomata **D** longitudinal section through conidiomata **E, F** conidiophores and conidiogenous cells **G** conidia. Scale bars: 1 mm (**A**); 200 µm (**B–D**); 10 µm (**E–G**).

##### Materials examined.

China, Beijing City, Fengtai Distinct, Beigong National Forest Park, 39°52'20.46"N, 116°7'47.60"E, from branches of *Euonymusjaponicus*, 12 Apr 2023, A.L. Jia & X.L. Fan (BJFC CF20230409, living culture CFCC 59450); Beigong National Forest Park, 39°52'20.46"N, 116°7'47.60"E, from branches of *Malus* ‘*American*’, 12 Apr 2023, A.L. Jia & X.L. Fan (BJFC CF20230410, living culture CFCC 59475); Century Forest Park, 39°49'43"N, 116°14'27"E, from branches of Acerpictumsubsp.mono, 18 May 2023, A.L. Jia & Y.X. Li (BJFC CF20230411, living culture CFCC 59471; BJFC CF20230412, living culture CFCC 59536).

##### Notes.

*Cytosporahaidianensis* was first introduced by Zhou et al. (2020) and which was isolated from *Euonymusalatus* in Beijing, China. This species has numerous locules with a central column of ostiolar tissue (Zhou et al. 2020). In this study, four isolates grouped together with *C.haidianensis* in ML and BI trees (ML/BI = 100/1). Therefore, they are identified as *Cytosporahaidianensis*. The current study extends its host range to *Buxusmegistophylla*, *Malus* ‘*American*’ and Acerpictumsubsp.mono.

#### ﻿ Cytospora pinea

Taxon classificationFungiDiaporthalesValsaceae

A.L. Jia & X.L. Fan sp. nov.

8CC4E63C-B1AC-5B41-A7BF-BB3F684C056F

850895

Fig. 8

##### Etymology.

Named after the host genus on which it was collected, *Pinus*.

##### Typification.

China, Beijing City, Fengtai Distinct, Lotus Pond Park, 39°53'27.64"N, 116°18'49.21"E, from branches of *Pinusbungeanae*, 9 Feb 2023, X.L. Fan (holotype BJFC CF20230413, ex-holotype living culture CFCC 59521; 39°53'27.21"N, 116°18'49.56"E, from branches of *Pinusbungeanae*, 9 Feb 2023, X.L. Fan (paratype BJFC CF20230415, ex-paratype living culture CFCC 59523).

##### Description.

***Sexual morph***: not observed. ***Asexual morph*: *Conidiomata pycnidial***, immersed in bark, scattered, nearly flat, slightly erumpent through the bark surface in a large area, with multiple locules. ***Conceptacle*** absent. ***Ectostromatic disc*** light brown to black, inconspicuous, circular to ovoid, with one ***ostiole*** per disc. ***Ostiole*** black, conspicuous, 150–200 μm diam. ***Locules*** numerous, irregular, subdivided frequently by invaginations with common walls, 980–1130 µm diam. ***Conidiophores*** borne along the locules, hyaline, branched at the base, in the middle or unbranched, thin-walled, 15–22 × 1.5–2.5 μm (av. = 18 ± 2.3 × 2 ± 0.3 μm, n = 30), embedded in a gelatinous layer. ***Conidiogenous cells*** enteroblastic, phialidic, sub-cylindrical, 3–7.5(–8) × 1–2 μm (av. = 4.5 ± 1.4 × 1.6 ± 0.3 μm, n = 50), tapering towards apices; arranged in rosettes. ***Conidia*** hyaline, allantoid, eguttulate, smooth, aseptate, thin-walled, 3.5–5 × 1–2 μm (av. = 4.3 ± 0.5 × 1.4 ± 0.2 μm, n = 50).

##### Culture characteristics.

Cultures on PDA are initially white, growing slowly up to 2 cm in diam. after 3 d and becoming yellowish after 7–10 d. Colonies thin with a uniform texture, lacking aerial mycelium, entirely covering the 6 cm Petri dish after 14 d, with a regular edge. After 30 d, *pycnidia* irregularly distributed on culture surface.

**Figure 8.**
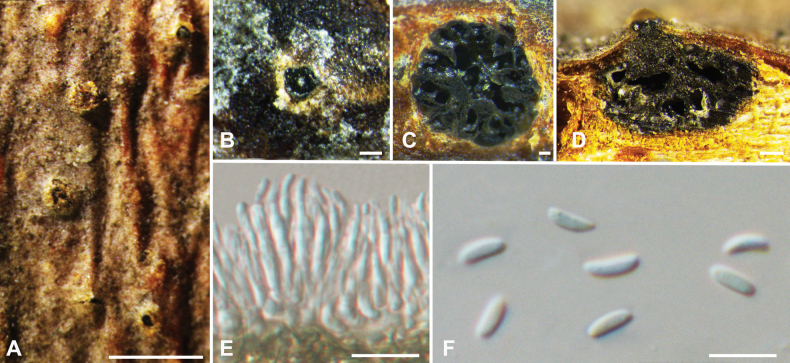
*Cytosporapinea* from *Pinusbungeanae* (BJFC CF20230413) **A, B** habit of conidiomata on branch **C** transverse section through conidiomata **D** longitudinal section through conidiomata **E** conidiophores and conidiogenous cells **F** conidia. Scale bars: 2 mm (**A**); 200 µm (**B, D**); 100 µm (**C**); 10 µm (**E, F**).

##### Additional materials examined.

China, Beijing City, Fengtai Distinct, Lotus Pond Park, 39°53'26.87"N, 116°18'43.46"E, from branches of *Pinusbungeanae*, 9 Feb 2023, X.L. Fan (BJFC CF20230414, living culture CFCC 59522; BJFC CF20230416, living culture CFCC 59524).

##### Notes.

*Cytosporapinea* is associated with canker disease of *Pinusbungeanae* in China. *Cytosporapinea* is close to *C.bungeanae* in the phylogenetic diagram (Fig. 2) and was isolated from the same host species *Pinusbungeanae* (Fan et al. 2020). It can be distinguished from *C.bungeanae* by smaller conidiophores (3–7.5(–8) × 1–2 vs. 15–27(–30) × 1.5–2 μm in *C.bungeanae*) and smaller locules (980–1130 vs. (1150–)1220–1480(–1600) μm in *C.bungeanae*). Furthermore, *Cytosporapinea* has a black conspicuous ostiole per disc, whereas the ostiole of *C.bungeanae* is inconspicuous. Phylogenetically, there are differences of 76/344 in the *act* region and 7/811 in the *tef1-α* gene with gaps.

#### ﻿ Cytospora sorbariae

Taxon classificationFungiDiaporthalesValsaceae

A.L. Jia & X.L. Fan sp. nov.

97AE52B0-5BFD-5FCD-8911-267F3FC6EA79

850896

Fig. 9

##### Etymology.

Named after the host genus on which it was collected, *Sorbaria*.

##### Typification.

China. Beijing City, Fengtai District, Beijing Garden Expo, 39°52'35.65"N, 116°11'4.02"E, from branches of *Sorbariasorbifolia*, 7 Apr 2023, A.L. Jia & X.L. Fan (holotype BJFC CF20230417, ex-holotype living culture CFCC 59445); 39°52'35.43"N, 116°11'4.62"E, from branches of *Sorbariasorbifolia*, 7 Apr.2023, A.L. Jia & X.L. Fan (paratype BJFC CF20230419, ex-paratype living culture CFCC 59529).

##### Description.

***Sexual morph***: not observed. ***Asexual morph*: *Conidiomata pycnidial*** immersed in the bark, scattered, erumpent through the surface of bark in a large area, with multiple locules. ***Conceptacle*** absent. ***Ectostromatic disc*** brown to black, circular to ovoid, erumpent through the surface of bark in a large area, conspicuous when mature, 160–300 µm in diam., with one or two ostioles per disc. ***Ostioles*** grey to black, at the same or slightly above the level of the disc surface, 50–85 µm in diam. Locules numerous, subdivided frequently by invaginations with common walls, circular to ovoid, 550–750 µm in diam. ***Conidiophores*** hyaline, unbranched, approximately cylindrical, 14–18 × 1–1.5 µm. ***Conidiogenous cells*** enteroblastic, phialidic. ***Conidia*** hyaline, elongate-allantoid, smooth, aseptate, 5.5–7.5 × 1.5–2.5 (av. = 6.5 ± 0.7 × 2 ± 0.3, n = 50) µm.

##### Culture characteristics.

Cultures on PDA are initially white, growing fast up to cover the 5.5 cm Petri dish after 3 d, becoming vinaceous buff after 7–10 d. Colonies are flat with a uniform texture, lacking aerial mycelium. Colony margin regular. After 30 d, *pycnidia* distributed irregularly on surface.

**Figure 9.**
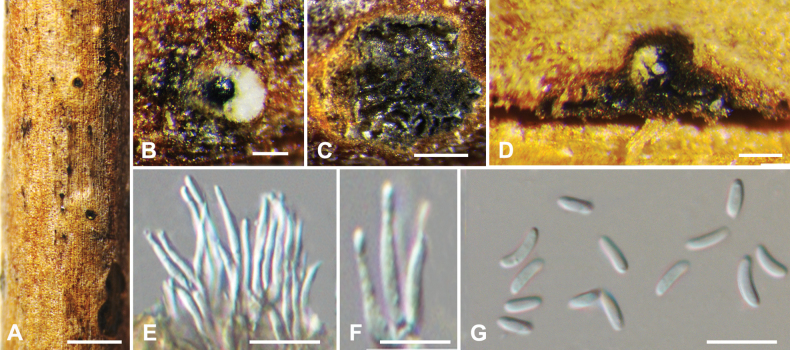
*Cytosporasorbariae* from *Sorbariasorbifolia* (BJFC CF20230417) **A, B** habit of conidiomata on branch **C** transverse section through conidiomata **D** longitudinal section through conidiomata **E, F** conidiophores and conidiogenous cells **G** conidia. Scale bars: 1 mm (**A**); 100 µm (**B–D**); 10 µm (**E–G**).

##### Additional materials examined.

China. Beijing City, Fengtai District, Beijing Garden Expo, 39°52'35.10"N, 116°11'4.31"E, from branches of *Sorbariasorbifolia*, 7 Apr 2023, A.L. Jia & X.L. Fan (BJFC CF20230418, living culture 59443; BJFC CF20230420, living culture 59530).

##### Notes.

*Cytosporasorbariae* is associated with canker disease of *Sorbariasorbifolia* in the current study. It can be identified by having conidiomata with a column lenticular tissue in the centre and its distinct disc of stromata on branches. Additionally, the four strains are phylogenetically separated from all other available strains included in this study. The clear multi-gene phylogram placed it in a distinct clade with high support (ML/BI = 100/1, Fig. 2).

## ﻿Discussion

The present study identified seven *Cytospora* species (*C.ailanthicola*, *C.albodisca*, *C.euonymina*, *C.fengtaiensis* sp. nov., *C.haidianensis*, *C.pinea* sp. nov. and *C.sorbariae* sp. nov.) from symptomatic branches and twigs associated with canker and dieback disease. This study represents an investigation of *Cytospora* species associated with canker disease in Fengtai District, Beijing and included a comprehensive analysis of DNA sequence data to compare the novelties with known *Cytospora* species.

In recent years, the study of *Cytospora* species on a particular host has received much attention from experts. For example, Jiang et al. (2020) identified six *Cytospora* species on Chinese chestnut (*Castaneamollissima*) which proved that *Cytospora* canker is a common disease on chestnut trees. Lin et al. (2023a) revealed the presence of *Cytospora* species from *Populus* in China and confirmed *Cytosporaailanthicola*, *C.chrysosperma*, *C.paratranslucens* and *C.sophoriopsis* as pathogens by pathogenicity tests. In this study, *Cytospora* species has a high diversity on *Malusspectabilis* and *Euonymusjaponicus* (*Cytosporaalbodisca* and *C.haidianensis*). There are many studies about *Cytospora* related to *E.japonicus*, while few studies on *Malusspectabilis* have been recorded (Lin et al. 2023b). Therefore, many varieties of *Malusspectabilis* associated with *Cytospora* species need a systematic study and their pathogenicity is required to be confirmed in the future.

*Cytospora* included both generalist pathogens and specialist pathogens (Lawrence et al. 2018). Most *Cytospora* species have been discovered in a wide range of hosts (Adams et al. 2005, 2006; Lawrence et al. 2018; Norphanphoun et al. 2018; Fan et al. 2020). In this study, *Cytosporasorbariae* and *C.fengtaiensis* were introduced as two new species from the single host species, so more exhaustive sampling from other regions of the world is needed in future studies for a clear elucidation of their host ranges and distribution.

In this article, seven species, associated with *Cytospora* disease, were identified in Fengtai District, Beijing. A targeted prevention and treatment strategy is needed to be drawn up. The occurrence of *Cytospora* canker and dieback diseases can be minimised by removing dead and dying branches in the dry season and maintaining susceptible trees as strong as possible. Moreover, the occurrence of *Cytospora* canker diseases is affected by the environment, distribution and transmission (Fan et al. 2015b), which may act as potential inoculum sources for other hosts in natural and artificial environments.

This study focused on *Cytospora* species in Fengtai District of Beijing, an attractive location with a high richness of fungal species (Zhu et al. 2018b, 2019). The descriptions and molecular data of *Cytospora* in this study could provide a resource for future studies in this genus and lay the foundation for the future investigation of canker disease caused by *Cytospora* species.

## Supplementary Material

XML Treatment for Cytospora ailanthicola

XML Treatment for Cytospora albodisca

XML Treatment for Cytospora euonymina

XML Treatment for Cytospora fengtaiensis

XML Treatment for Cytospora haidianensis

XML Treatment for Cytospora pinea

XML Treatment for Cytospora sorbariae

## ﻿Additional information

### Author ORCIDs

Aoli Jia https://orcid.org/0009-0004-0265-5454

Xinlei Fan https://orcid.org/0000-0002-4946-4442

### Data availability

All of the data that support the findings of this study are available in the main text.
